# Routine chemoprophylaxis for deep venous thrombosis in Indian patients: Is it really justified?

**DOI:** 10.4103/0019-5413.33680

**Published:** 2007

**Authors:** Ashutosh P Mavalankar, Darshan Majmundar, Shubha Rani

**Affiliations:** Dr. Jivraj Mehta Hospital, Ahmedabad -380 007, India; * B. V. Patel PERD Centre, Ahmedabad -380 007, India

**Keywords:** Deep venous thrombosis, pulmonary embolism, thromboprophylaxis

## Abstract

**Background:**

Venous thromboembolism (VTE), which consists of deep vein thrombosis (DVT) and pulmonary embolism, is a potentially fatal disease. According to the Western literature, DVT of lower limb veins is one of the most common complications following total hip and knee arthroplasty and surgeries for lower limb fractures. Very few studies have been published from India on the subject and very little is known about the true incidence of the condition. The issue has acquired greater significance in Indian subjects in recent times as there is a manifold increase in the number of joint replacement surgeries and surgeries for lower limb fractures. There are no clear guidelines regarding the prophylaxis for VTE for Indian patients.

**Materials and Methods:**

We carried out a prospective study to determine the incidence of DVT. Present study included 125 patients undergoing total knee and hip joint arthroplasty and surgeries for fractures of the lower limb over a three-year period. All the patients underwent duplex ultrasonography between the seventh and 14^th^ postoperative day. No mechanical or chemical form of DVT prophylaxis was used.

**Results:**

Only nine patients (7.2%) showed sonographic evidence of DVT and the majority of them resolved without treatment. There was no case of pulmonary embolism.

**Conclusion:**

DVT following total joint arthroplasty and surgery for lower limb fractures in Indian patients is not as common as reported in the Western literature. A high level of suspicion and close clinical monitoring is mandatory, routine chemoprophylaxis is perhaps not justified in every patient undergoing lower limb surgery in our opinion. More trials involving a larger number of patients and at multi centers, in future, would be required to confirm the findings of our study.

Venous thromboembolism (VTE), which consists of deep vein thrombosis (DVT) and pulmonary embolism (PE), is a potentially fatal disease. Long-term sequelae, particularly post phlebitic syndrome (PPS) are frequent and often disabling. The initial aim of treatment of DVT is prevention of thrombus extension and PE.

DVT of lower limb veins is one of the most common complications following total hip and knee arthroplasty and surgery for fractures of the lower limb. Without any mechanical or pharmacologic prophylaxis, asymptomatic DVT develops in 40-60% of the patients undergoing total hip and knee arthroplasties.^1^ Proximal DVT develops in 15-25% and a fatal pulmonary embolism develops in 0.5-2% patients.^2^ Many risk factors like advanced age, fracture of the pelvis, hip, femur or tibia, prior venous thromboembolic disease, obesity etc. have been identified for the occurrence of venous thromboembolic disease.^3^

The majority of the studies have been conducted and published from the western countries where DVT is more commonly seen. On the other hand, very few papers have been published from this part of the world where DVT was, until recently, considered to be a rarity. We, therefore, have to follow the western literature for the guidelines on thromboprophylaxis for patients undergoing major lower limb surgery. Though some of the recent studies published from other Asian countries have shown that DVT is not a rarity in Asian patients as was thought earlier.^4^,^5^ We searched “Pubmed” for the published studies on the subject from India, we could find very few studies.^6^–^8^ Parakh *et al.*^9^ observed that very few studies are available to assess the prevalence of PE in Asian countries and found that PE occurs frequently in Indian patients with symptomatic DVT. Parakh, Kakkar *et al.*^10^ in their review article have stated that the Indian perspective on this topic is lacking due to the non-availability of published Indian data. They believe that venous thrombosis may occur in more than 50% of patients undergoing surgical procedures, particularly those involving the hip and knee.

The issue has become even more relevant today as the number of total joint arthroplasties and trauma surgery for lower limb fractures has seen a manifold increase in the last few years with very few Indian surgeons offering some kind of thromboprophylaxis to their patients undergoing major lower limb surgery. Also, there are medico-legal implications of not subjecting patients undergoing lower limb surgery to some kind of thromboprophylaxis, as some people consider this an act of negligence. We, therefore, decided to undertake this prospective study at our institution to determine the incidence of DVT in Indian patients undergoing total joint replacement arthroplasty and surgery for fractures of lower limb by duplex ultrasonography. This method was selected because it has a sensitivity of 100% and a specificity of 97%. It is a safe, effective and quick technique for diagnosing venous thrombosis in patients. It is well accepted by both patients and staff and is without any inherent risks.^11^

## MATERIALS AND METHODS

A prospective study of 125 consecutive cases of total hip and knee arthroplasty and surgeries for fractures of lower limb carried out over a three-year period from June 2003 to June 2006. A prior consent was obtained from all the patients and the study was approved by the Ethical Committee of the Hospital. There were 45 male patients (Average age-60 years, Range 25-90 years) and 80 female patients (Average age-62 years, Range 29-94 years). Out of these 125 patients, 59 patients underwent total knee replacement, 11 underwent total hip replacement, 26 underwent internal fixation or hemiarthroplasty for proximal femoral fracture, 11 underwent interlocking tibial/femoral nailing and 18 underwent internal fixation for other lower limb fractures. All the operations were carried out under regional (spinal ± epidural) anesthesia. Any known risk factor associated with occurrence of DVT like past history of DVT, presence of varicose veins, obesity, malignancy etc. was recorded. Other variables e.g., age, sex, height, weight, presence of any medical problems etc. were also documented for a possible correlation with the occurrence of DVT. Note was also made of the clinical evidence of DVT before subjecting the patient to Doppler study. No mechanical or chemical form of DVT prophylaxis was given to any of the patients.

A strict protocol for mobilization of the patients following surgery was observed and all the patients except one patient who had fixation of acetabular fracture were mobilized 48h after surgery. All the patients underwent duplex ultrasonographic assessment of both the lower limbs between the seventh and 14^th^ postoperative day. The Doppler assessment included examination of bilateral common femoral, superficial femoral, popliteal, anterior tibial and posterior tibial veins. They were assessed for flow, visualized thrombus, compressibility and augmentation. A diagnosis of DVT was made where there was visualization of thrombosis, absence of flow, lack of compressibility or lack of augmentation. The thrombus was classified as distal if it involved the calf veins only and as proximal if it involved the popliteal or a more proximal vein. Patients who had both a proximal and a distal thrombus were classified as having proximal thrombosis.

The patients who developed postoperative distal venous thrombosis diagnosed by Doppler examination were not subjected to any form of thrombolytic treatment. They were, however, kept under close clinical observation. The patients who showed evidence of proximal DVT were subjected to standard thrombolytic treatment in the form of infusion of unfractionated Heparin at the rate of 1000 IU per hour. Activated partial thromboplastin time (APTT) was closely monitored and was maintained at 1.5-2.5 times control. Tab. Warfarin-5 mg per day was commenced simultaneously and international normalized ratio (INR) was monitored every two days. When therapeutic level of INR (between 2 and 3) was achieved, Heparin infusion was discontinued. Warfarin was continued for about three to four months. A repeat Doppler study was performed in all these patients within a week of the first positive Doppler study to make sure that there was no further propagation of the thrombus.

## RESULTS

Out of 125 patients, DVT was detected in nine patients (three males and six females) with the average age 74 years (63-94 years). The distribution of thrombus in different veins is shown in Table 1. Out of these nine patients, only three patients had evidence of proximal DVT while the remaining six patients showed distal DVT.

**Table 1:** Distribution of thrombus in the lower limb veins detected by duplex ultrasonography (n=9)

Sex/ age	Operative procedure	Ant. tibial vein	Post. tibial vein	Popliteal vein	Superf. femoral vein	Common femoral vein	Calf veins
M/90	A.M. hemiarthroplasty		+	+	+	+	+
F/65	Total knee arthroplasty		+				+
M/65	ORIF #Acetabulum		+			+	+
F/94	D.H.S. fixation		+				+
F/63	A.M. hemiarthroplasty				+		
M/68	D.H.S. fixation		+				
F/76	A.M. hemiarthroplasty						+
F/63	Total knee arthroplasty		+				
F/75	Total hip arthroplasty				+	+	

The DVT positive cases were classified according to the surgical procedure carried out [Table 2]. We observed that DVT developed more commonly following surgery for femoral neck fractures compared to other surgeries like total knee replacement (n=2, 3.4%) and total hip replacement (n=1, 9.1%). Occurrence of DVT was highest following surgery for proximal femoral fractures (five out of 26 patients, i.e. 19.2% of 26 patients undergoing surgery for femoral neck fractures developed DVT). It was lowest in the patients undergoing total knee replacement (two out of 59 patients, i.e. only 3.4% of 59 patients undergoing total knee replacement developed postoperative DVT).

**Table 2:** Occurrence of deep vein thrombosis with various operative procedures (n=9) operative procedure

(Total- 125)	No. of DVT positive cases(9)
Total knee replacement (59)	2 (3.4%)
Total hip replacement (11)	1 (9.1%)
Proximal femoral fracture (26)	
D.H.S. (18)	
A.M. Hemiarthroplasty (8)	5 (19.2%)
Miscellaneous trauma surgery (29)	1 (3.4%)

Out of nine positive cases for DVT, only two had clinical signs of DVT like calf swelling and tenderness. Also, no association was found between the occurrence of DVT and body mass index (BMI) as shown in Table 3.

**Table 3:** Body mass index

	Patient population (n =125)	DVT positive cases (n = 9)
Range	19.1-32.5	20.2-28.6
Average	24.7	24.33

All the patients with evidence of deep venous thrombosis (both proximal and distal) underwent repeat Doppler study one week after the detection of thrombus to rule out proximal extension of the thrombus. Six patients with distal DVT did not show evidence of further propagation of thrombus on repeat Doppler study. In fact, there was resolution of thrombosis in all the patients. All the three patients who showed evidence of proximal DVT at the time of first Doppler examination and who were subjected to standard thrombolytic treatment did not show any propagation of thrombus. None of the patients developed clinically evident pulmonary embolism while in hospital or during the first six weeks following surgery.

## DISCUSSION

Until recently, deep vein thrombosis following lower limb surgery was considered to be a rarity in Asian patients. Dhillon *et al.*,^4^ in a prospective study of 88 patients from Singapore without any prophylaxis, reported that 62.5% of the patients demonstrated venographic evidence of DVT. They further suggested that the present practice of withholding routine prophylaxis against thromboembolism in Asian patients undergoing high-risk orthopedic procedure should be reconsidered. Ko *et al.*^12^ in a prospective study of 80 “low-risk” Chinese patients undergoing total knee arthroplasty (TKA) and total hip arthroplasty (THA) showed 27-31% incidence of postoperative deep vein thrombosis detected by duplex sonography. They concluded that patients who are labeled “low-risk” for DVT actually had a significant risk and suggested that the current practice of providing prophylaxis to only patients deemed at “high risk” should be revised. However, Jain *et al.*,^6^ reported a very low incidence of DVT following TKA and THA in Indian patients. Only two patients in their series of 106 patients from Northern India undergoing THA and TKA showed duplex sonographic evidence of proximal DVT. Similarly, Bagaria *et al.*^7^ reported 6.12% incidence of DVT and 0.6% incidence of PE in their prospective study of 147 patients undergoing major orthopedic surgery of lower limb without any prophylaxis. They concluded that DVT has a lower incidence in Indian patients as compared with other ethnic groups. Agarwala *et al.*,^8^ by using contrast venography as a diagnostic tool for DVT, however, reported 60% incidence of DVT in patients not receiving chemoprophylaxis and 43.2% incidence of DVT in patients receiving prophylaxis following major lower limb surgery in their study of 94 patients. Eighty-three per cent of these patients had distal DVT and there was not a single case of pulmonary embolism.

In our study, we have observed that out of 125 patients operated for major lower limb surgery, nine patients (7.2%) demonstrated sonographic evidence of DVT out of which three patients (2.4%) had a proximal deep venous thrombosis and six patients (4.8%) had a distal deep venous thrombosis. There was not a single case of pulmonary embolism. These results are comparable to those published by Jain *et al.* who had 1.9% rate of proximal DVT in their series of 106 patients without a single case of pulmonary embolism and with those published by Bagaria *et al.*,^7^ who reported 6.2% incidence of DVT. It is rather interesting that though all our patients came from the state of Gujarat, one of the Western Indian states with a different demographic pattern compared to the North Indian patients included in study by Jain *et al.*, the incidence of proximal DVT is around 2%. So, it may not be inappropriate to assume that the same incidence would be applicable to the Indian population in general. However, more extensive prospective trials are required to be conducted in different parts of the country to substantiate or reject this hypothesis. Our results, however, are not in agreement with those published by Agarwala *et al.*,^8^ who reported 60% incidence of DVT in their patients not receiving any form of chemoprophylaxis. We believe that this difference is because of the difference in the diagnostic modality used in both studies. While Agarwala *et al.* used contrast venography, which is the gold standard, for diagnosis of both proximal and distal DVT, we employed duplex sonography for detection of postoperative DVT. Though this is an established diagnostic modality, many authors have questioned its ability to diagnose asymptomatic calf thrombi as it may miss 20% of isolated calf DVT.^13^,^14^ So, isolated calf thrombi in some of the patients may not have been picked up by Doppler examination in our study. Also, Agarwala et al do not seem to have elaborated on the venographic evidence of DVT in their patients. This is because nonfilling of contrast in deep veins on phlebography is claimed to be an indirect sign of DVT by some authors but rejected by others. Bjorgell *et al.*^15^ showed that isolated nonfilling of the posterior tibial and/or deep muscle veins of the calf found by phlebography may be an indirect sign of DVT but is equally commonly caused by other pathological conditions like edema, bleedings, ligament and muscle ruptures, Baker cysts, and superficial thrombophlebitis or arises without any detectable explanation.

Though age is uncertain as a risk factor, we found an increasing evidence of thrombosis with greater age. All our DVT positive patients were in the age group of 63-94. As regards known risk factors responsible for occurrence of postoperative DVT as mentioned earlier, we found that except one female diabetic patient who developed extensive proximal thrombosis following surgery, none of the other eight patients had a known risk factor for development of DVT.

We did not find any correlation between the presence of clinical signs of thrombosis and sonographic evidence of DVT. Out of nine, only two patients (22%) had clinical features suggestive of DVT. This confirms unreliability of physical signs in the diagnosis of postoperative DVT as shown by Stulberg *et al*.^16^

It is also known that the prevalence of thrombophilia, which is a hereditary or acquired condition that predisposes individuals to thromboembolic events like myocardial infarction, deep vein thrombosis, pulmonary embolism etc, is much less in the Indian patients compared to their counterparts in the western countries. Resistance to activated protein C (APC) is the most common inherited risk factor for venous thrombosis. Most cases of APC resistance are caused by Factor V Leiden mutation. This mutation is the most frequent genetic disposition for thrombophilia and DVT and has a carrier rate of 2.9% in the Dutch population, 5% in Poland and only 1.3% in Punjab, India.^17^ This fact could be responsible for low incidence of DVT and PE in Indian patients.

All the patients included in our study had their surgery performed under spinal/epidural anesthesia. Though it is claimed by many authors that this can reduce the incidence of DVT, it is difficult to draw this conclusion from our study as all the patients underwent surgery in spinal anesthesia and there was no control to compare the incidence of DVT in patients undergoing surgery under general anesthesia.

We also observed that that DVT developed more commonly following surgery for femoral neck fractures compared to other surgeries like total knee replacement and total hip replacement. Occurrence of DVT was highest following surgery for proximal femoral fractures (five out of 26 patients, i.e., 19.2% of 26 patients undergoing surgery for femoral neck fractures developed DVT). It was lowest in the patients undergoing total knee replacement (two out of 59 patients, i.e., only 3.4% of 59 patients undergoing total knee replacement developed postoperative DVT). This is contrary to the findings of Agarwala *et al.*,^8^ who observed 55-60% of their patients operated for total knee replacement showing venographic evidence of DVT.

As six out of nine patients had distal DVT, we decided not to treat them with anticoagulation. Kakkar *et al.,*^18^ have shown that popliteal and femoral clots are precursors to pulmonary emboli. This is in sharp contrast to tibial and peroneal clots, which are of little clinical significance with respect to symptomatic emboli. Many other authors have also recommended treating asymptomatic distal DVT by close clinical observation and serial Duplex ultrasound study.^19^–^21^

Low molecular weight heparin (LMWH) which is currently recommended to be one of the preferred drugs for thromboprophylaxis has many potential disadvantages like increase in the total cost of treatment and bleeding complications. McNally *et al.*,^22^ have also stated that increased incidence of bleeding complications like excessive bruising around the wound and increased wound bleeding or hematomas with the use of LMWH has prevented their routine use in joint replacement, as was the case with unfractionated heparin in the past. We, therefore, wonder how far we are justified in subjecting all the patients undergoing arthroplasty or fracture surgery in the lower limb to routine chemoprophylaxis which could increase the risk of hematoma formation, infection, a re-operation and a prolonged hospital stay and put more burdens on the already stretched financial resources of the patient.

From our study and those by other Indian authors,^6^,^7^ it appears that DVT and PE in Indian patients is a fairly low-incidence problem. The sample size of 125 patients in our study seems to be reasonable, as the sample size required for the estimation of incidence rate (of value 0.07) is 123 with an error of 0.045. Also, if thromboprophylaxis is important for all the patients, at least a few patients would have required that. In our study, none of the patients were subjected to chemoprophylaxis. Though the number is rather small, it is not too small to say that thromboprophylaxis is not important for all the patients. Further studies are required to confirm the findings of this research.

## CONCLUSION

We believe that though there is enough evidence in the Western literature to advocate routine thromboprophylaxis for patients undergoing total joint replacement and surgery for fractures of lower limb, there is not yet enough evidence to justify the same for Indian patients undergoing major lower limb surgery. Though it is perhaps not appropriate to make any definite recommendation about chemoprophylaxis only on the basis of our research, we strongly agree with Gillespie *et al.*^23^ and advocate that the orthopedic surgeons should use pharmacological prophylaxis only for the high-risk patients (advanced age, past history of DVT, presence of varicose veins, obesity, malignancy, immobilization, etc.) in whom the potential benefits clearly appear to outweigh the risks. However, a close clinical monitoring with a high level of suspicion for DVT and pulmonary embolism must be exercised. A duplex sonography should be preferably carried out on all the elderly and high-risk patients undergoing total joint arthroplasty or surgery for lower limb fractures between the seventh and 14^th^ postoperative day and a repeat ultrasonography should be performed on all the positive cases to rule out proximal propagation of thrombus. Trials involving a larger number of patients in future are required to confirm findings of this research which would help resolve the dilemma for the orthopedic surgeons in India whether or not to subject their patients undergoing lower limb surgery to chemoprophylaxis for DVT and PE.
